# The Role of Epigenetic Control of Mitochondrial (Dys)Function in MASLD Onset and Progression

**DOI:** 10.3390/nu15224757

**Published:** 2023-11-12

**Authors:** Valerio Caputo, Giovanni Tarantino, Silvano Junior Santini, Giovanna Fracassi, Clara Balsano

**Affiliations:** 1 Department of Life, Health and Environmental Sciences-MESVA, School of Emergency-Urgency Medicine, University of L’Aquila, 67100 L’Aquila, Italy; v.caputo91@gmail.com (V.C.); silvanojunior.santini@univaq.it (S.J.S.); giovanna.fracassi@graduate.univaq.it (G.F.); 2 F. Balsano Foundation, Via Giovanni Battista Martini 6, 00198 Rome, Italy; 3 Department of Clinical Medicine and Surgery, Federico II University of Naples, 80138 Naples, Italy; tarantin@unina.it

**Keywords:** NAFLD, mitochondrial dysfunction, bioactive compounds, epigenetics, DNA methylation, non-coding RNA, liver

## Abstract

Metabolic dysfunction-associated steatotic fatty liver disease (MASLD), a novel definition for NAFLD, represents one of the most common causes of liver disease, and its incidence is increasing worldwide. It is characterized by a complex etiopathogenesis in which mitochondrial dysfunction exerts a pivotal role together with alteration of lipid metabolism, inflammation, and oxidative stress. Nutrients and bioactive compounds can influence such mechanisms so that changes in diet and lifestyle are regarded as important treatment strategies. Notably, natural compounds can exert their influence through changes of the epigenetic landscape, overall resulting in rewiring of molecular networks involved in cell and tissue homeostasis. Considering such information, the present review aims at providing evidence of epigenetic modifications occurring at mitochondria in response to natural and bioactive compounds in the context of liver (dys)function. For this purpose, recent studies reporting effects of compounds on mitochondria in the context of NAFLD/MASLD, as well as research showing alteration of DNA methylation and non-coding RNAs-related circuits occurring at liver mitochondria, will be illustrated. Overall, the present review will highlight the importance of understanding the bioactive compounds-dependent epigenetic modulation of mitochondria for improving the knowledge of MASLD and identifying biomarkers to be employed for effective preventative strategies or treatment protocols.

## 1. Introduction

Nonalcoholic fatty liver disease (NAFLD) is reckoned as one the most frequent causes of liver disease. NAFLD was very recently re-named metabolic dysfunction-associated steatotic fatty liver disease (MASLD). This novel definition focuses on the presence of hepatic steatosis together with at least one of five cardiometabolic criteria based on BMI, fasting serum glucose levels, blood pressure, plasma triglycerides, and HDL/cholesterol levels. Nevertheless, it should be stressed that the discrepancies between NAFLD and MASLD are all in all negligible [1]. Based on such consideration and on the fact that most of the herein illustrated studies still depict the disease as NAFLD, we will employ such a definition throughout the manuscript.

Like the obesity pandemic, NAFLD trends are skyrocketing all over the world. The estimated global incidence of NAFLD is 47 cases per 1000 of the population, with a global prevalence of NAFLD among adults of 32% [2]. According to a nationwide, matched cohort study including patients having undergone liver biopsy and followed for a median of 14.2 years, the excess mortality associated with NAFLD was primarily from extra-hepatic cancer, followed by cirrhosis, cardiovascular disease, and hepatocellular carcinoma [3].

Mechanisms are various and complex. NAFLD is characterized by fat accumulation in hepatocytes, the so-called hepatic steatosis, due to an imbalance between lipid acquisition and lipid disposal, which are regulated through four major pathways: uptake of circulating lipids, de novo lipogenesis (DNL), fatty acid oxidation, and export of lipids in very low-density lipoproteins (VLDL) [4].

Insulin-mediated stimulation of DNL, leading to an increased conversion of glucose to fatty acids, gains more and more importance as a regulator of intrahepatic triglyceride content in individuals with NAFLD [5]. Consistently, insulin resistance (measured by Homeostasis Model Assessment-Insulin Resistance, HOMA-IR) has been reported as a triggering condition of mortality in NAFLD [6,7]. 

Notably, adiponectin exerts an important protective role by reducing hepatic and systematic insulin resistance as well as attenuating liver inflammation and fibrosis [8]. In particular, adiponectin was found to enhance the expression and activity of PPARG Coactivator 1 Alpha (PGC-1α) by inducing Ca^2+^ uptake through its receptor AdipoR1 in murine muscle cells. PGC-1α is a well-known regulator of mitochondrial biogenesis by activating key regulators of mitochondrial metabolism (including NRF1, MEF2C, and PPARα) [9]. Accordingly, an increased number of functional mitochondria was found, while the silencing of AdipoR1 led to an overall mitochondrial impairment associated with oxidative stress and insulin resistance-like phenotype [10].

The interaction between mitochondria (dys)function and insulin resistance has also been consistently reported in the context of NAFLD [11] and mitochondrial dysfunction is being increasingly regarded as a pivotal mechanism leading to steatosis. A recent study supported the impairment of mitophagy as an early event in liver from mito-Keima FBVN mice fed with Western Diet as models of steatosis, detecting a loss of 25% of mitophagy after six weeks of the diet [12].

Interestingly enough, the inner mechanisms involving mitochondria likely are at the basis of the drug-induced hepatotoxicity, associating the two apparently distant diseases [13]. Considering such increasing evidence on the relevance of mitochondrial dysfunction for NAFLD pathogenesis, the present review will illustrate the evidence concerning modulation of mitochondrial function by natural and bioactive compounds, particularly focusing on related epigenetic changes occurring at mitochondria.

## 2. Mitochondrial Dysfunction and NAFLD/MASLD

Indeed, mitochondria represent fundamental hubs of cell metabolism and energy, as well as of molecular signaling, immune functions, and cell viability. These organelles are involved in glucose and lipid catabolism by means of the tricarboxylic acid (TCA) cycle and β-oxidation pathway, respectively. Therefore, mitochondria play a fundamental role in nutrients and natural compound metabolism, from which they produce ATP by means of electron transport chain and oxidative phosphorylation process [14]. Notably, ATP exerts a well-known fundamental role in supporting the biochemical reactions that ensure the homeostasis of cells and, ultimately, of the entire organism. Considering such role, it is not surprising that dysfunction occurring at mitochondria may be detrimental on a plethora of diseases [15].

On this subject, the respiratory chain represents a source of ROS which can affect proteins, lipids, and even DNA functions, ultimately leading to the disruption of cell physiology due to excessive oxidative stress [16].

Indeed, different conditions may contribute to an increased ROS production, including dietary components (such as fatty acids), hyperglycemia-related disorders, and even changes in the intestinal microbiome [17,18,19]. A high concentration of fat may enhance the mitochondria-related biochemical processes, especially the β–oxidation and, initially, the respiration, leading to an increase of ATP production that, in a feedback loop, further exacerbates the release of ROS. In this way, mitochondria biogenesis can be dramatically impaired, with a reduced number of mitochondria functionally active, since they can be characterized by a lower abundance of mtDNA and a strongly decreased activity of oxidative enzymes. Thus, as mentioned above, the loss of mitochondrial function together with the oxidative stress may be considered typical hallmarks of NAFLD and markers of progression [17,18].

Supporting this, it is important to remark that cytoplasmatic sources of ROS, namely the NADPH oxidases (NOXs), whose abundance in liver cells is closely related to inflammation and immune responses, have been reported to contribute to the transition from NAFLD to NASH [20].

Precedent data pointed out that peripheral insulin resistance (IR), increased fatty acid beta oxidation, and hepatic oxidative stress are present in both NAFLD and the more severe form, i.e., nonalcoholic steatohepatitis (NASH). NASH alone was associated with mitochondrial structural defects [11].

On the contrary, in a more recent work, hepatic mitochondrial dysfunction has been shown to precede the development of NAFLD and IR in sedentary, hyperphagic, obese Otsuka Long-Evans Tokushima fatty rats. Measures of hepatic mitochondrial content and function including beta-hydroxyacyl-CoA dehydrogenase activity, citrate synthase activity, and immunofluorescence staining for mitochondrial carbamoyl phosphate synthetase-1, progressively worsened at 40 weeks. This evidence suggests that initial, but also progressive, mitochondrial dysfunction contributes to the natural history of obesity-associated NAFLD [21].

There is agreement among experts in accepting the association between different food group intakes/dietary patterns and NAFLD [22], as well as the interaction of such components with mitochondrial function. For instance, the latest NHANES data showed that higher dietary choline, which can be found in whole eggs, fish, and meat, was associated with a lower risk of NAFLD in both American females and males [23]. Of note, a choline-deficient diet is strictly associated with impairment of the VLDL secretion and ß-fatty acid oxidation due to the low availability of phosphatidylcholine (a related metabolite important for the correct formation of VLDL particles and the solubilization of bile salts), which, as above mentioned, leads to an increased fat content in the liver [24]. Moreover, findings exist that indicate that the disruption of mitochondrial membrane potential is an upstream event in choline deficiency (CD)-induced apoptosis, and mitochondrial dysfunction plays a key role in mediating CD-induced apoptosis in cultured rat hepatocytes (CWSV-1 cells) [25]. Choline deficiency was also found to lead to altered DNA methylation and gene expression of critical genes involved in DNA mismatch repair, resulting in increased mutation rates [26]. Furthermore, endocrine-disrupting chemicals might play a role in NAFLD initiation and evolution with mechanisms including an imbalance between lipid influx/efflux in the liver, liver inflammation, and epigenetic reprogramming and mitochondrial dysfunction [27]. Undoubtedly, taking into account the illustrated evidence, mitochondrial dysfunction represents a fundamental mechanism underlying NAFLD etiopathogenesis. Therefore, the role that bioactive molecules, nutrients, and dietary active compounds plays in influencing NAFLD onset and progression, by modulating the mitochondrial gene expression profiles and function, may be of paramount importance. On this subject, epigenetics, which encompasses molecular mechanisms able to shape cell profiles in response to environmental factors, likely exert a fundamental role in mediating the action of compounds on mitochondria metabolism in the context of liver homeostasis when focusing on NAFLD. 

## 3. Bioactive Compounds Exerting Effects on Mitochondrial Function and Metabolism in the Context of NAFLD/MASLD

Concerning NAFLD, mitochondria dysfunction has been characterized as a pathogenetic feature, both in its onset and progression [11], as above mentioned, and it can also result from xenobiotic impacts, such as drugs, exposure, and excessive alcohol use [28].

Therefore, it is not surprising that various research studies investigated how dietary and natural compounds could impact on mitochondria with the purpose of preventing or treating NAFLD. Of note, it ought to be remarked that, still to date, changes in diet and lifestyle represent one of the most important dedicated treatments [29], even though there are many drugs on the pipeline that are reckoned as good candidates to cure this very common liver disease [30].

That said, there is an unmet need to use natural products by many physicians to alleviate NAFLD on the basis of plenty of literature data [31]. Herein, we will illustrate some recent works reporting effects on mitochondrial function due to the activity of bioactive compounds (Table 1).

Among the most investigated natural compounds, polyphenols represent a class of natural antioxidants widely studied for their healthy effects, also considering their presence in a wide range of dietary components, including fruits, vegetables, grains, as well as tea and coffee [39].

Interestingly, a preclinical study focused on investigating the utility of Resveratrol against NAFLD in vitro, exploiting HepG2 cell lines, as well as in vivo, evaluating samples from high fat diet (HFD)-treated rats. Among the positive effects, the authors reported a peroxisome proliferator-activated receptor alpha (PPARα)-dependent improvement of the enzymatic activities of mitochondrial complexes I and IV of the respiratory chain and antioxidant enzymes, including catalase (CAT) and superoxide dismutase 1 (SOD1) in both liver tissues and HepG2 cells [32].

Resveratrol was also evaluated in male newborn piglets affected by intrauterine growth retardation (IUGR), which is considered an important risk factor for developing NAFLD. Importantly, such animals displayed vacuolation of hepatocytes and disorganization of liver parenchyma. Also in this case, resveratrol was found to significantly enhance the activity of mitochondrial I complex as well as complexes II and III, and ATP levels. Interestingly, mtDNA copy number was evaluated and reported to be increased by resveratrol treatment, thus suggesting a contextual improvement of mitochondrial biogenesis. Considering the link of IUGR with NAFLD and the fact that the employed animals displayed NAFLD-like hepatic injuries, authors suggested that resveratrol could be prioritized for NAFLD treatment [33].

Similarly, another study evaluated Taurisolo, a nutraceutical derived from grapes extremely enriched in Resveratrol, gallic acid, and procyanidins, for the protective effects both on HuH7 hepatoma cells and HFD mice. In particular, hepatoma cells treated with Taurisolo displayed an increased activity of mitochondrial respiration of 1.20, measured by cytofluorimetry, with respect to non-treated cells. Such data were further confirmed in the Taurisolo-treated mice recapitulating NAFLD features, whose liver samples displayed significantly increased levels of mitochondrial metabolites (citrate, malate, and acetil-coA) as well as ATP (*p* < 0.001) [40]. 

Research has also been performed on other polyphenols derived from olive oil and olives, such as hydroxytyrosol and Oleuropein, and the influence on mitochondrial metabolism with the aim of counteracting liver steatosis. In particular, hydroxytyrosol was reported to moderately restore the expression and the activity of PPARα and to increase the expression of antioxidant enzymes, including glutathione S-transferase (GST) and nuclear factor erythroid 2-related factor 2 (NRF2), in liver from HFD mice, leading to a less inflammatory environment and thus to a recovery of steatotic features [34]. Such rescue could also be due to a possible effect on mitochondria given by the compound action on PPARα and NRF2. As a matter of fact, PPARα has been recently found to up-regulate the activity of mitochondrial complexes of the respiratory chain [32]. 

Moreover, NRF2 activity has been found able to enhance the oxygen consumption and to act as a transcriptional activator for NRF1 and PGC1-α, leading to an increased biogenesis in murine models [41,42]. 

Accordingly, hydroxytyrosol has been recently evaluated in combination with docosahexaenoic acid, an omega 3 fatty acid, in murine models. The study allows the detection in the related liver samples of a significantly increased activity of complexes I and II and of ATP levels, as well as NAD+/NADH ratio in HFD mice co-administered with the two compounds compared to non-administered mice [35]. 

Other studies evaluated Oleuropein and found a comparable positive effect on mitochondria function. In particular, Oleuropein was reported to enhance the expression of *superoxide dismutase 2 (SOD2)* on liver samples from HFD mice as well as restoring antioxidant defense related to mitochondria by acting on CAT. In addition, similar to Valenzuela et al., 2017, Oleuropein was able to up-regulate *NRF2* and *GST* [36]. Interestingly, Oleuropein also was independently able to induce autophagy by gender-specific mTOR activation in liver of HFD mice [43].

Of note, an up-regulation of cytochrome *C oxidase copper chaperone 17 (COX17)* was observed in such samples after Oleuropein treatment [37]. COX17 is a component of the complex IV for which it acts as a copper chaperone, able to transport copper from cytosol to the Mitochondrial Cytochrome C Oxidase I and II (MT-CO1/2) [37,44].

Moreover, a study evaluated the effects of hydroethanolic extracts of Guava leaves (enriched in Quercetin) on liver samples from young rats provided with fructose, as a model resembling pediatric NAFLD. The research found various pathways and metabolic activities restored in the fructose-fed rats treated with the Guava leaves extracts, including a response to oxidative stress and recovery of mitochondrial activity. In particular, authors found that the activity related to mitochondrial complexes I, II, and IV as well as of ATP synthase were significantly increased (*p* < 0.05), thus supporting the potential application of such extracts for clinical purposes [38].

The illustrated studies strongly support both direct and indirect effects of bioactive and natural compounds on the mitochondria function and metabolism. The reported dysregulation of mitochondrial factors, both at transcript and protein levels, may suggest that such compounds could alter mitochondrial epigenetic background. 

It is important to remark that the described studies evaluating bioactive compounds targeting mitochondria in NAFLD are mainly focused on models based on cell lines or animals. This raises the need of performing investigations on more advanced disease models, such as organoids that are able to recapitulate the structure and function of the tissue, including the different cell types and thus, representing optimal tools for recapitulating human disease features. In this way, it will be possible to obtain robust biomarkers or novel potential therapeutical targets to be further investigated at a clinical level.

## 4. Bioactive Compounds Influencing Epigenetics at Mitochondria in Liver

### 4.1. Investigations on Mitochondrial Epigenetic Marks of NAFLD

Epigenetics encompasses molecular factors and signals able to shape gene expression profiles at different levels in response to inner and external stimuli (including age, lifestyle, and disease status) without modification of the DNA sequence. In particular, DNA methylation and hydroxymethylation, histone modification, and non-coding RNA (nc-RNA)-dependent regulation are important epigenetic mechanisms able to modulate cell function and thus to influence the homeostasis of tissues [45]. 

As a matter of fact, DNA methylation at the promoter sequences of genes is well-known to be associated with transcriptional repression, whereas noncoding-RNAs (nc-RNAs) including long non-coding (lnc-RNAs), microRNAs (miRNAs), and circular RNA (circ-RNAs) are involved in complex molecular circuits with other RNAs and proteins, leading to changes in transcriptome and protein abundance and functions. Histone modifications, including lysine acetylation or variable methylation, are strictly linked to chromatin open or close conformation. The modelling of epigenetic modifications is mediated by specific factors, able to establish, modify, or erase such signals. The activity of proteins such as DNA methyltransferases/demetylases or histone acetylases/deacetylases (HAT, HDACs) can result in changes in epigenetic profiles that can in turn lead to rewiring of gene expression patterns or interactome [45,46].

Based on such activity, it is not surprising that epigenetic signals have been increasingly investigated for their involvement in human pathological conditions. On this subject, epigenetics has been evaluated for its influence on dysmetabolism, including lipid metabolism alteration, immunity, and inflammation, which are known mechanisms linked to NAFLD and its progression [47,48]. Importantly, evidence supports that alteration of epigenetic landscape may also contribute to mitochondrial dysfunction [48]. 

It is important to remark that the mitochondrial genome displays peculiar features, such as the absence of protective histones that account for a different spatial organization with respect to nuclear DNA [49,50].

Research efforts have, therefore, mainly focused on mitochondrial DNA (mtDNA) methylation profiles and the effect of post-transcriptional regulation mediated by nc-RNAs.

Concerning the DNA methylation patterns in the pathogenesis of NAFLD, a study investigated such modification in NAFLD patient-derived liver biopsies, focusing on the progression from steatosis to NASH. In particular, this study evaluated the DNA methylation levels of specific regions of the mitochondrial genome, namely the *D-loop* (the non-coding region that acts as a promoter for mitochondrial genes), the *mitochondrially encoded NADH:ubiquinone oxidoreductase core subunit 6 (MT-ND6)*, and *mitochondrially encoded cytochrome C oxidase I (MT-CO1)*. Only *MT-ND6* was found to be slightly significantly hypermethylated in NASH samples with respect to the simple steatosis (*p* < 0.05). Such a pattern was found to be significantly associated with a decreased transcription of the correspondent gene in NASH patients and the hypermethylation and downregulation patterns both correlated with the severity of liver fibrosis, as a histological parameter [51]. Interestingly, *MT-ND6* codes for a subunit of complex I of the mitochondrial respiratory chain. As afore mentioned, the dysfunction of this chain is strictly linked with ROS excessive production.

Concerning non-coding RNAs, several microRNAs targeting mitochondrial factors or miRNAs encoded by mitochondrial genome have been identified with an effect on mitochondrial function and metabolism in case of dysregulation [52] and a wide range of microRNAs able to affect mitochondrial homeostasis has been studied in in vitro and in vivo models of NAFLD. Notably, miR-146a, miR-29a, miR-34a, miR-181, and miR-873-5p are among the microRNAs reported to exert detrimental effects, when dysregulated, on mitochondrial biogenesis and mtDNA abundance, oxygen consumption rate and ROS production, ATP production, and fatty acid oxidation in NAFLD [53,54,55,56,57,58]. 

Epigenetic events occurring in mitochondria as a response to bioactive and natural compounds represent, still to date, a land to be explored, especially in the context of NAFLD. Now, considering the outcomes of the above-mentioned studies, investigating the potential effects of bioactive compounds may be extremely promising in light of improving knowledge on disease features and identifying novel promising druggable targets. On this subject, there is evidence that rewiring of nc-RNA-mediated circuits and alteration of DNA methylation patterns of the mitochondrial genome may be mediated by bioactive compounds in liver.

### 4.2. Bioactive Compounds Influencing Mitochondrial DNA Methylation Patterns in Liver

**Figure 1:**
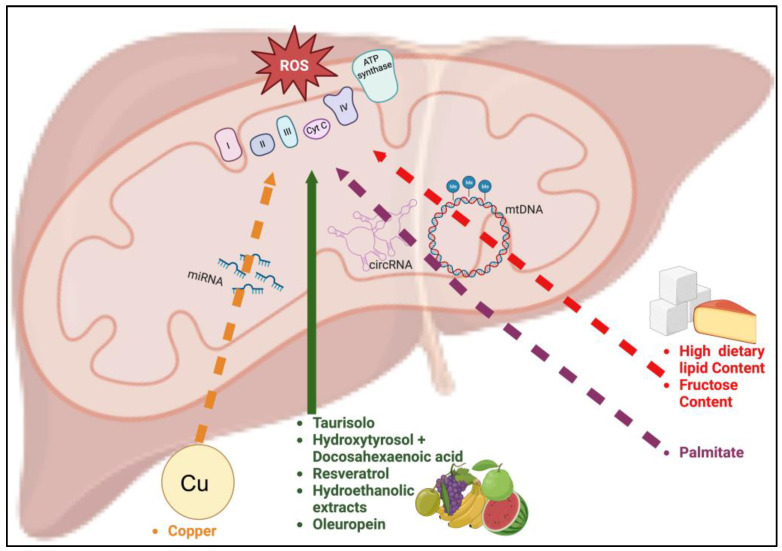
Illustration recapitulating the routes of natural compounds influencing mitochondrial (dys)function. Bioactive compounds with positive effects on mitochondrial function are illustrated (green route, solid line). The picture also illustrates those compounds affecting mitochondrial function through local epigenetic modulation (DNA methylation patterns and alteration of local nc-RNAs levels). The routes related to high dietary lipids and fructose (red route), palmitate (purple route), and copper (orange route) are reported. Dashed lines indicate that the compound increased oxidative stress and mitochondrial dysfunction.

Research efforts have been made to unveil the effect of nutrients in modulating the DNA methylation patterns characterizing hepatic mitochondria on animal models (Table 2, Figure 1). 

A study was aimed at evaluating the effect of four dietary lipids, namely sunflower oil, palmitic acid, olive oil, and perilla oil, on the methylation patterns occurring at mtDNA in the liver of fishes (yellow croakers) [59] (Table 2).

In this way, authors found significantly higher methylation levels at *arginine transfer RNA (MT-TR)* and *NADH:ubiquinone oxidoreductase core subunit 4L (MT-NAD4L)* in samples provided of olive and perilla oils compared to those administered with fish oil. Conversely, *mitochondrially encoded 12S ribosomal RNA (MT-RNR1)* was found to be significantly hypomethylated in the case of an olive-oil-based diet with respect to a fish-oil-based one. 

Intriguingly, authors also tested muscle and adipose tissues, although the obtained methylation patterns resulted in being specific to liver. This may suggest the occurrence of tissue-related events. The study also evaluated the oxidative stress status and activity of anti-oxidant enzymes. On this subject, authors found an increased expression of HIF1-α in samples provided with olive and perilla oils, which were the same ones with the highest methylation occurring at *MT-TR* and *MT-NAD4L*. The methylation patterns appeared to be correlated to the transcriptional alteration of the correspondent genes. Furthermore, other mitochondrial genes, including *mitochondrially encoded NADH:ubiquinone oxidoreductase core subunit 3(MT-ND3*), *MT-ND6*, and *mitochondrially encoded 16S ribosomal RNA (MT-RNR2*) were analysed and found as differentially expressed depending on the diet. Of note, *MT-ND3* and *MT-NAD4L*, together with *MT-ND6*, encode subunits of complex I of the mitochondrial respiratory chain. In keeping with such transcriptional dysregulation, the activity of the assembled complex I was significantly impaired in olive and perilla oil groups (*p* < 0.05). Moreover, samples from the olive oil group were characterized by a higher hydroxyl radical scavenging activity, while this group together with the perilla oil one displayed increased activity of CAT and Glutathione peroxidase (GPx) [59]. 

Another study by the same group evaluated the effect of low, moderate, and high content of crude lipids within diets on the mitochondrial metabolism and epigenetic status. Also in this case, authors employed yellow croakers. This research found that high lipid content was significantly associated with hypermethylation of the *D-loop* region and hypomethylation of *MT-RNR1*. Authors also found a slight modulation of MT-ND6 methylation which, however, did not reach a statistical significance. An increased abundance of mtDNA in case of high lipid-based diet was observed, as well. However, no significant alteration of mitochondrial genes mRNA was observed in this group, which, according to authors, may suggest the influence of other factors at transcriptional and post-transcriptional levels [60]. 

Overall, these two studies suggest that the type and quantity of dietary lipids may differentially affect mtDNA methylation. Undoubtedly, the outcomes of these studies deserve to be deeply verified in mammals and human-derived models and then evaluated for translational purposes. Whether the modulation of mitochondrial DNA methylation profiles represents an initial event or a consequence of the oxidative dysregulation given by lipid uptake remains to be elucidated, as well. Moreover, given the role of lipid dysmetabolism in the pathogenesis of NAFLD, further investigation on the influence on mtDNA methylation patterns could unravel intriguing insights on pathogenetic mechanisms and help in identifying disease biomarkers to be exploited for more precise medical protocols.

In addition to dietary fatty acids, high sugar consumption may also contribute to dysmetabolism and to NAFLD. On this subject, evidence has been found on a potential modulation of mtDNA methylation by high fructose concentration. In particular, research investigated the effects of a high level of fructose in the diet on mtDNA methylation, exploiting, in this case, liver samples from rats. Authors reported a global hypomethylation of the mtDNA in liver from rats fed with a high fructose content (*p* < 0.01) and, accordingly, also an increased transcription of various mtDNA genes, namely *MT-ND3*, *mitochondrially encoded NADH:ubiquinone oxidoreductase core subunit 4 (MT-ND4)*, *MT-ND4L*, *mitochondrially encoded NADH:ubiquinone oxidoreductase core subunit 5 (MT-ND5)*, *MT-ND6*, *mitochondrially encoded ATP synthase membrane subunit 6 and 8 (MT-ATP6*, *MT-ATP8)*, *mitochondrially encoded cytochrome B (MT-CYB)*, and *MT-CO1/3* (overall *p* < 0.05). 

Furthermore, an increased mtDNA copy number was found. Interestingly, authors also evaluated the hydroxymethylation at a cytosine residues (5hmc) rate, finding it to be highly significantly reduced in the context of high fructose content (*p* < 0.01) and related with the methylation percentage [61]. 

According to the authors, fructose, which in a past study by them was found to induce NAFLD-like liver injuries in rats, may elicit compensatory responses (i.e., mechanisms for increasing mitochondrial enzyme transcription) by mitochondria to counteract such injuries [64]. 

### 4.3. Bioactive Compounds Influencing Post Transcriptional Regulation and nc-RNA-Mediated Circuits in Liver

The action of bioactive compounds on nc-RNAs in the context of NAFLD has started being evaluated (Figure 1). A very recent study investigated the effect of copper on microRNAs transcribed from the mitochondrial genome in livers from chicken and embryo-derived hepatocytes, exposed and non-exposed to the copper. Importantly, excessive uptake of this metal was reported as detrimental for a plethora of human chronic disorders, including NAFLD. In particular, high serum concentrations have been found associated with cirrhosis and hepatocellular carcinoma in NAFLD patients [65,66]. In the work by Zhong et al., a significant overexpression of gga-miR-12294-5p was detected both in mitochondria of treated liver samples and cells (*p* < 0.05). Such a signature was further investigated by means of co-treatment/transfection of copper and miR-12294-5p-mimic as well as with luciferase assay. In this way, the authors identified CDGSH Iron Sulfur Domain 1 (Cisd1) as a mRNA target for the miRNA. Notably, this gene encodes an iron-containing protein located at the outer mitochondrial membrane that is involved in regulation of electron transport and oxidative status [62,67]. 

The study reported that the inhibition of Cisd1 by miR-12294-5p binding, in case of treatment with copper and miRNA overexpression, was able to impair redox homeostasis and mitochondrial quality control. In fact, the exacerbation of oxidative stress was shown by a reduced membrane potential and a decreased concentration of mitochondrial mediators of redox processes (namely SOD2, TXN2, PRDX3, GPX4, both at a transcript and protein level) in cells transfected with the miR-12294-5p mimic and treated with copper. Moreover, the mitochondrial quality control was impaired, considering the occurrence in the same conditions of the downregulation of factors involved in mitochondrial biogenesis and dynamics, including fusion and clustering, such as Mitofusin 1 and 2 (MFN1, MFN2), PGC-1α as well as of Nuclear Respiratory Factor 1 (NRF1), Nuclear Factor Erythroid 2-Related Factor 2 (NRF-2), TFAM and Transcription Factor B1, Mitochondrial (TFB1M), and overexpression of both Dynamin 1-like (DRP1) and its interactor mitochondrial fission factor (MFF), which in turn are involved in mitochondrial fission [62]. Overall, this study highlights the rewiring of fundamental processes for the mitochondrial and cell homeostasis caused by the modulation of micronutrients on miRNA-target genes’ circuits, supporting further research on mammals and human experimental models recapitulating NAFLD features. 

A special attention has been recently given to another class of non-coding RNAs, namely the circ-RNAs. Importantly, circ-RNAs appeared to be versatile molecules. As a matter of fact, such nc-RNAs are able to act as miRNAs’ sponge, inhibiting their function, to directly bind proteins, to regulate transcription or splicing events; they also have been reported to be translated into proteins or peptides. Given this plethora of functions, the dysregulation of circ-RNAs expression is likely to lead to dramatic changes in cell and overall tissue homeostasis. Many research efforts reported an alteration of circRNAs in the context of neoplasies, including hepatocarcinoma in vitro models [68,69].

Evidence of modulation of circ-RNAs located within hepatic mitochondria due to lipids comes from a study that found that the steatohepatitis-associated circRNA ATP5B regulator (SCAR) circ-RNA levels were highly significantly lower in liver fibroblasts obtained from normal subjects when treated with palmitate compared to non-treated. Furthermore, this lower abundance was associated with ROS overproduction and reduced mitochondrial membrane potential [63]. The study, which performs elegant experiments aimed at showing the therapeutic potential of modulating SCAR levels to restore mitochondrial function in NASH patients, strongly supports further investigation at a clinical level for this circ-RNA and sheds light on the clinical potential of modulating this class of nc-RNAs in the context of NAFLD.

## 5. Future Perspectives and Conclusions

The above-mentioned studies on the epigenetic modulation due to the action of natural compounds show how such research may unveil promising insights into NAFLD. Dissecting the epigenetic control of mitochondrial (dys)function and understanding how external stimuli may modulate it will lead to a deeper knowledge of disease mechanisms and thus to important achievements for therapeutical purposes. In particular, it will be interesting to investigate whether the compounds acting on mitochondrial function in steatotic liver (Table 1) are able to exert such effects through modulation of the local epigenetic landscape.

In addition, the NAFLD-associated epigenetic signatures and agents mentioned in Section 4.1 could be targeted by dietary compounds in order to improve the mitochondrial function and thus the cell and tissue homeostasis. Such research will shed light on potential positive effects of dietary components and may be useful for tailored diet to counteract the progression of liver injuries. Importantly, the methylation patterns occurring at mitochondrial regions in response to bioactive compound in liver tissue as well as the “mito-ncRNAs” able to shape the local transcripts expression (Table 2) deserve investigation in proper models of NAFLD, in order to evaluate their relationship with the pathogenesis or progression and understand how their modulation can be exploited against the disease.

Other important insights might come from the investigation of compounds able to interact with lnc-RNAs. Indeed, such nc-RNAs are often involved in molecular circuits together with miRNAs and proteins, and thus may globally influence both gene expression and protein activity, contributing to different disease phenotypes [70]. Supporting a potential role in NAFLD, the alteration of several lnc-RNAs levels was detected in both patients’ blood and liver [71].

Of note, there is evidence of lnc-RNAs acting within mitochondria that, in response of nutrients uptake, can be modulated and can lead to modification of the local metabolism. One interesting example is represented by the glucose-dependent downregulation of lnc-RNA GAS5 in breast cancer experimental models. In particular, such low GAS5 levels were correlated with increased mitochondrial activity (including increased TCA activity and ATP production) and cell proliferation [72]. Instead, GAS5 was found upregulated in HFD mice, suggesting a potential contribution to steatosis. Consistently, the downregulation of this lnc-RNA led to a decreased degree of steatosis in murine livers [73]. 

Therefore, investigating the influence of glucose/carbohydrates on modulating GAS5-miRNAs-target genes’ circuits could represent promising research to dissect the contribution to mitochondrial (dys)function in NAFLD. 

As illustrated in the present review, DNA methylation patterns in liver mitochondria may be modulated in response of nutrients and bioactive compound and thus represent promising candidate biomarkers of disease or therapeutical targets upon further research in NAFLD. In this context, intriguing insights may also come from the investigation of DNA hydroxymethylation patterns at mtDNA, as already highlighted in Section 4.2. In particular, hydroxymethylation at cytosine (5-hmc) represents an intermediate and stable product of the de-methylation process performed by TET enzymes and it appears to be strictly tissue-specific. Overall, it is widely considered a marker of proliferating cells and, in fact, a lower rate of 5-hmc has been detected in several cancer types [74]. Notably, a study evaluated the role of 5-hmc in NAFLD pathogenesis. In particular, this research was conducted on liver biopsies obtained from NAFLD/NASH and near-normal liver histology (NNLH) patients as controls. The authors reported a positive correlation between 5-hmC and mtDNA copy number (*p* < 0.01) as well as a weak correlation with *PGC1-α* mRNA (*p* = 0.04). Therefore, authors propose that 5-hmc could contribute to NAFLD by altering the mitochondrial biogenesis [75]. Overall, investigating how bioactive compound may modulate lnc-RNAs or influence DNA methylation/hydroxymethyation could represent a strategy to prevent steatosis in at-risk patients or counteract NAFLD progression.

Intestinal dysbiosis is increasingly regarded as a pathogenetic factor of NAFLD, since the alteration of the microbiome has been detected in affected patients, also highlighting differences depending on the disease progression [76,77,78].

Intriguingly, there is evidence of cross-talks between intestinal microbiota and liver mitochondria [19,76]. On this subject, an altered gut-liver axis function, which involves translocation of bacterial products into portal circulation as a result of gut leakiness, has been linked to increased production of ROS and inflammation in the liver [79]. Considering that dietary components are able to influence microbiome composition, the interaction of bioactive compounds with intestinal microbiota may unveil indirect effects contributing/counteracting NAFLD pathogenesis as well as progression to NASH. In particular, it could be interesting to assess how bioactive compounds may influence the release into the circulation of different abundance of pathogen-associated molecular patterns (PAMPs) and bacterial products (including also postbiotic metabolites such trymethylamine and short fatty-acids) and evaluate whether such products, beside influencing inflammatory responses [79,80], also elicit epigenetic modulation of mitochondrial function in liver. 

In conclusion, it is nowadays clear how mitochondrial function is fundamental for the homeostasis of liver and how mitochondria can be sensitive in response of external factors, including bioactive compounds and nutrients that can act through epigenetic signals for shaping their functions. Thus, understanding the molecular mechanisms by which such compounds may modulate mitochondrial function will not only improve the knowledge of NAFLD and its progression, but also may provide novel and hopefully more effective targets to be employed in the clinical practice. On this subject, the translation of mitochondrial epigenetic marks could be facilitated by applying advanced computation methods able to integrate such marks with other biomarkers and clinical data, in order to obtain a more comprehensive picture of the disease and of the patient. This could be relevant in the future for improving the decision-making process, not only related to preventative strategies based on diet and lifestyle for at-risk of NAFLD individuals, but also for predicting and counteracting the progression to NASH or cirrhosis and for more effective therapeutical strategies.

## Figures and Tables

**Table 1:** Bioactive compounds modulating mitochondrial function and their activity in NAFLD.

Bioactive Compound	Effect Dose	Cell/Animal Model	Mitochondrial Targets/Effects	Reference
Resveratrol	40 μM	HepG2 hepatocarinoma cells treated with palmitic acid and liver samples from HFD male Sprague Dawley rats	Increased activity of complexes I and IV of mitochondrial respiratory chain Increased activity of CAT and SOD1	Huang et al., 2020, [32]
	300 mg/kg of feed	Liver samples from male newborn pigs	Increased [ATP] levels Increased activity of complexes I, II, and III of mitochondrial respiratory chain Increased [mtDNA]	Cheng et al., 2021, [33]
Taurisolo	800 mg/L	HuH 7.5, human hepatoma cells 7, clone 5, and liver samples from HFD male and female C57BL/6 mice	Increased activity of mitochondrial respiration Increased [ATP] levels Increased metabolites related to mitochondrial biochemical reactions	Valenzuela et al., 2017, [34]
Hydroxytyrosol + Docosahexaenoic acid	Docosahexaenoic acid: 50 mg/kg of body weight per day; Hydroxytyrosol: 5 mg/kg of body weight per day	Liver samples from HFD male C57BL/6J mice	Increased activity of complexes I and II of mitochondrial respiratory chain Increased [NAD+/NADH], [ATP] levels	Ortiz et al., 2020, [35]
Oleuropein	0.03%	Liver samples from HFD male and female C57BL/6J mice	Upregulation of SOD2, CAT, GST, and NRF2	Santini et al., 2020, [36]
		Liver samples from HFD male and female C57BL/6J mice	Upregulation of COX17	Santini et al., 2022, [37]
Hydroethanolic extracts of Guava leaves	500 mg/kg of body weight per day + 15% fructose	Primary hepatocytes derived from weaned male albino Wistar rats	Increased activity of complexes I, II, IV, and ATP synthase of mitochondrial respiratory chain	Sharma et al., 2022, [38]

The compound, the dose at which the effect has been detected, the employed in vitro or in vivo models, as well as the effects on mitochondria have been reported for each study. Abbreviations: HFD, high fat diet; CAT, Catalase; SOD1, Superoxide dismutase 1; ATP, Adenosine triphosphate; mtDNA, mitochondrial DNA; NAD+, Nicotinamide adenine dinucleotide; NADH, Nicotinamide adenine dinucleotide + Hydrogen (reduced form of NAD+); SOD2, Superoxide dismutase 2; GST, glutathione S-transferase; NRF2, nuclear factor erythroid 2-related factor 2; COX17, cytochrome C oxidase copper chaperone 17.

**Table 2:** Bioactive compounds able to influence mitochondrial epigenetics in liver.

Epigenetic Signal/Agent	Bioactive Compound	Effect Dose	Cell/Animal Model	Mitochondrial Epigenetic Signature	Reference
DNA methylation	Dietary lipids (olive oil, sunflower oil, palmitic acid, perilla oil and fish oil)	80 g/kg of body weigth	Liver samples from yellow croakers	Hypermethylation at MT-TR and MT-NAD4L in olive oil and perilla oil groups Hypomethylation at MT-RNR1 in olive oil group	Liao et al., 2015, [59]
	Dietary lipids abundance	Diets based on 6%, 12% and 18% crude lipid content	Liver samples from yellow croakers	Hypermethylation at D-loop region in high lipid content group Hypomethylation at MT-RNR1 in high lipid content group	Liao et al., 2015, [60]
	Fructose	20% fructose solution	Liver samples from male Sprague Dawley rats	Global mtDNA hypomethylation	Yamazaki et al., 2016, [61]
Nc-RNAs	Copper	330 mg/kg for liver samples, 100 μM for primary embryo hepatocytes	Liver samples and primary embryo hepatocytes from Arbor Acres broiler chicken	Overexpression of gga-miR-12294-5p/Inhibition of Cisd1	Zhong et al., 2023, [62]
	Palmitate	250 μM	Liver fibroblasts from normal subjects	Downregulation of SCAR circ-RNA	Zhao et al., 2020, [63]

The compound and the dose, which the effect has been detected at, the employed in vitro or in vivo models, as well as the resulting epigenetic signature on mitochondria have been reported for each study. Abbreviations: TR, arginine transfer RNA; MT-NAD4L, NADH:ubiquinone oxidoreductase core subunit 4L, MT-RNR1, mitochondrially encoded 12S ribosomal RNA; D-loop, displacement loop region; Cisd1, CDGSH Iron Sulfur Domain 1; SCAR, steatohepatitis-associated circRNA ATP5B regulator.

## Data Availability

The presented data are included in the manuscript.
